# Infodemiology on diet and weight loss behavior before and during COVID-19 pandemic in Indonesia: Implication for public health promotion

**DOI:** 10.3389/fnut.2022.981204

**Published:** 2022-09-28

**Authors:** Ira Dewi Ramadhani, Leny Latifah, Andjar Prasetyo, Marizka Khairunnisa, Yurika Fauzia Wardhani, Diah Yunitawati, Mochammad Fahlevi

**Affiliations:** ^1^Faculty of Public Health, Sriwijaya University, Palembang, Indonesia; ^2^Research Center for Public Health and Nutrition, National Research and Innovation Agency Republic of Indonesia, Jakarta, Indonesia; ^3^Regional Development Planning Agency, Magelang, Indonesia; ^4^Department of Management, BINUS Online Learning, Bina Nusantara University, Jakarta, Indonesia

**Keywords:** infodemiology, Google trends, loss weight, diet, health promotion

## Abstract

**Objective:**

This study set out to explore public interest through information search trends on diet and weight loss before and during the COVID-19 pandemic in Indonesia.

**Methods:**

The Google Trends database was evaluated for the relative internet search popularity on diet-related search terms, including top and rising diet-related terms. The search range was before and during the COVID-19 pandemic (April 2018 to January 2022) in the Indonesia region. We analyzed the Relative Search Volume (RSV) data using line charts, correlation, and comparison tests.

**Results:**

Search queries of “lose weight” was higher during the pandemic (58.34 ± 9.70 vs. 68.69 ± 7.72; p<0.05). No difference was found in diet-related searches before and after the pandemic. Public interest in the diet was higher after Eid al-Fitr (Muslims break fasting celebration day) and after the new year. Many fad diet (FD) terms were found on the top and rising terms.

**Conclusion:**

After Eid al-Fitr and the new year were susceptible times for promoting a healthy diet in Indonesia. Potential need found before those times for education in inserting healthy food among fatty and sugary menus related to holidays and celebrations. Higher interest in “lose weight” was relevant to heightened obesity risk during the social restriction and heightened COVID-19 morbidity and mortality due to obesity. The high interest for rapid weight loss through FD needs to be resolved by promoting healthy diets with a more captivating message and messenger, like consistently using top terms in the keywords of the official healthy diet guidance. Future research could explore the relationship between diet and other behavior or with non-communicable diseases.

## Introduction

The prevalence of obesity has increased rapidly in the last four decades. In 2016, adult obesity reached 13%, and overweight 39% (1). Rising Body Mass Index (BMI) is a major risk factor for cardiovascular disease, diabetes, musculoskeletal disorder, and several cancers (1, 2). Obesity and body dissatisfaction may lead to weight loss behavior (3, 4). Body shape and weight dissatisfaction encourage people to gather information for methods to attain the perceived ideal body image, which could eventually lead to unhealthy or unsafe behavior (5, 6). Body image problems are closely related to, but do not solely occur in, overweight or obese people. As has been widely studied, women and men with normal BMI status can experience body image problems (7, 8).

Undertaking an FD is one of the most popular ways to achieve an ideal body image. FDs are quick diets with limited types and amounts of nutrients or certain food combinations without clear scientific backgrounds (9, 10). Indonesia is a country with an increasing proportion of obesity, overweight, and body image problems, and is therefore prone to FD (3, 11–13). Therefore, FD types spread quickly and become famous through the internet, and usage increased.

Increasing internet usage has encouraged Google Trends (GT) use in many study fields globally, particularly for infodemiology. Infodemiology is a science related to consumer and public health informatics. Infodemiology studies the distribution and determinants of information through internet media. The process involves mining textual user-generated data from the internet, systematically aggregating and analyzing these textual, unstructured data, then presenting findings as graphs, tables, and/or maps (14). GT has successfully demonstrated the ability to detect outbreaks of influenza, as well as early detection of infectious diseases, and implementing Google Trends studies results in forecasting, surveillance, and monitoring purposes in various health sectors (14, 15). For example, a study on dengue disease found a surge in dengue disease-related public information-seeking behavior related to dengue outbreaks or rising cases (15). Health studies using GT have shown great potential for monitoring health behavior problems (16–19). Effective and practical strategies were needed to increase people's awareness of diet-related weight loss, mainly in this COVID-19 pandemic (18, 20, 21) which hit all aspects of daily life (22). Obesity became a great concern during the COVID-19 pandemic due to adverse outcomes and a greater risk of death (23). On the other hand, the way we fight the COVID-19 pandemic worsens the obesity pandemic (24). As social isolation and distancing lowered physical and social activities (22), preventive approaches were needed for obesity during the COVID-19 pandemic (23).

The number of internet users in Indonesia reached 202.6 million in early 2021, an increase of 27 million from the previous year (25). Internet usage for information updates and finding health information was also quite high. The enormous amount of user-generated data enhances the nutritional infodemiology potential for reviewing health behavior and developing health promotion in Indonesia (26). The ultimate aim of infodemiology is to inform public health and public policy (14).

Previous studies about weight loss behavior were conducted in four season countries and differentiated the trend of searches based on seasonality (18, 21). Indonesia lies along the equator, has a tropical climate, and different cultural background. The higher risk of obesity during social distancing during the COVID-19 pandemic was assumed to heighten the interest in weight loss and diet-related terms on web searches. No previous study related to weight control and weight loss behavior infodemiology in Indonesia connected it to promotion and prevention strategies.

Nutritional infodemiology research using massive data could become a resource to produce evidence-based policy. Using GT, this study will explore diet and weight loss behavior before and during the COVID-19 pandemic in Indonesia. We would analyze the pattern of weight loss and diet–related query search activities before and during the COVID-19 pandemic to understand the receptive time for dietary health promotion. It is also essential to explore public interest in diet and weight loss methods, to get a healthy nutrition promotion formulation that could captivate the public interest.

## Materials and methods

GT data were downloaded for analysis on the same date to minimize the bias (February 11, 2022) (15, 27). All searches used “all categories” and “web searches” (image, news, Google shopping, and YouTube searches) in “Indonesia.” GT data is in the form of RSV with weekly data and has a scale of 0–100. The number 0 indicated the most minor used keywords, and 100 showed the most used keywords. Weekly period data were transformed into monthly periods using the mean to facilitate the analysis. RSV mean usage was aimed to increase the reliability of GT data (28). GT data were downloaded in Comma Separated Value (CSV) format.

### Search strategy

The previous study determined the search term by face validity or common terms in the study area (16, 29, 30). This study selected the search terms based on common terms in the diet and weight loss behavior area. The chosen main search terms were “diet” and “weight loss” (“*diet*” and “*penurunan berat badan*” in Bahasa). The most frequently used search from the top and rising search terms by GT suggestion were also explored. The insignificant and unrelated queries were deleted (16). The queries provided by GT were aggregated, and the fluctuation of RSV (data stability) was analyzed.

### GT analysis

The GT data graphically represented the timeline analysis. We analyzed the similarity of patterns between keywords from the increase, peak, and decrease from April 2018 (23 months before the COVID-19 pandemic) to January 2022 (the last available data at the time of the analysis, 23 months during the COVID-19 pandemic). The trendline fluctuation was matched with some events that occurred in that period.

Shapiro-Wilk test was run for the normality test, while major and important peaks in RSV values were compared with annual means. Pearson correlation test (R) was used to assess the correlation among diet-related search terms. We declared strength correlation for *R*-value 0.7 (*p*< 0.05). The Independent *t*-test and Mann-Whitney test were employed to compare periods (before and during the COVID-19 pandemic) among topics.

## Results and discussion

### Results

Based on the GT top and raising queries related to the “diet” and “lose weight,” the data were classified into five main topics. The first topic was “diet-related queries,” and three other topics were related to FD, the last topic was a single query of “lose weight.” Among popular and trending topics, we identified 16 specific and FD-related queries. We clustered the FDs or specific diet topics into “fruit and vegetable related FD” and “public figure FD,” with the rest classified into “other FD.” The detailed queries of the four aggregate topics could be found in Table 1. This research showed that all the top and raising queries had the words “diet,” including the FD and specific diet.

**Table 1:** Description of the diet topic.

**Topic**	**Search terms**
Diet-related queries	“diet,” “healthy diet,” “quick diet,” “diet method,” “diet menu,” “food for diet”
Fruit and Vegetable FD	“fruit diet,” “natural diet,” “lemon diet,” “vegetables diet,” “plant based diet,” plum diet
Public Figure Diet	“tya ariestya diet,” “rina gunawan diet,” “ricky cuaca diet,” “iu diet,” “debm diet”^a^ “OCD diet”^b^
Other FD	“milk diet,” “keto diet,” “tea diet,” “rice diet,” “egg diet,” “flimty diet”

a “debm diet” stand for diet enak bahagia menyenangkan or a fun happy and delicious diet developed by RH Liembono, a social media influencer;

b “OCD diet” stands for obsessive corbuzier's diet a diet developed by Deddy Corbuzier, a public figure.

**Figure 1:**
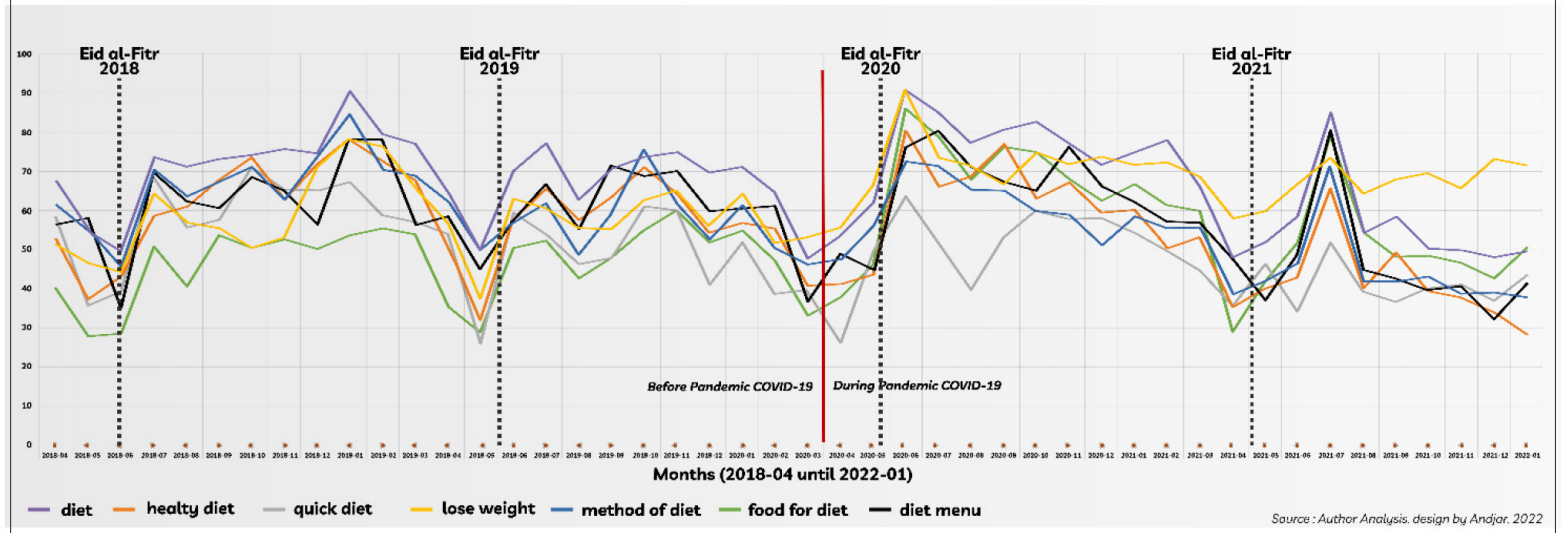
Timeline according to the topic of diet-related queries and periods (before and during the COVID-19 pandemic).

The timeline analysis for diet-related queries and weight loss before and during the COVID-19 pandemic showed a similar pattern (Figure 1), also indicated by the significant correlation between diet-related queries (Table 2). All diet-related search terms showed a similar pattern, including “lose weight” until December 2020 (Figure 1). Afterward, “lose weight” showed a more stable trend until January 2022. It explains why the “lose weight” query tended to have weaker correlations than other diet-related queries (Table 2). Statistical analysis comparing diet-related queries, FD, and “lose weight” before and during the pandemic showed that only “lose weight” was significantly higher.

**Table 2:** Correlation between diet-related queries.

	**Healthy diet**	**Quick diet**	**Loss weight**	**Diet method**	**Food for diet**	**Diet menu**
Diet	0.9223^***^	0.7548^***^	0.5027^**^	0.8820^***^	0.7668^***^	0.9055^***^
Healthy diet		0.7701^***^	0.3869^**^	0.8713^***^	0.6534^***^	0.8516^***^
Quick diet			0.3473^*^	0.7668^***^	0.4595^**^	0.6436^***^
Lose weight				0.2710	0.7590^***^	0.3514^*^
Diet method					0.4876^**^	0.8313^***^
Food for diet						0.6550^***^

Statistical results correspond to Pearson correlation:

* p < 0.05,

** p < 0.01,

and

*** p < 0.001.

**Figure 2:**
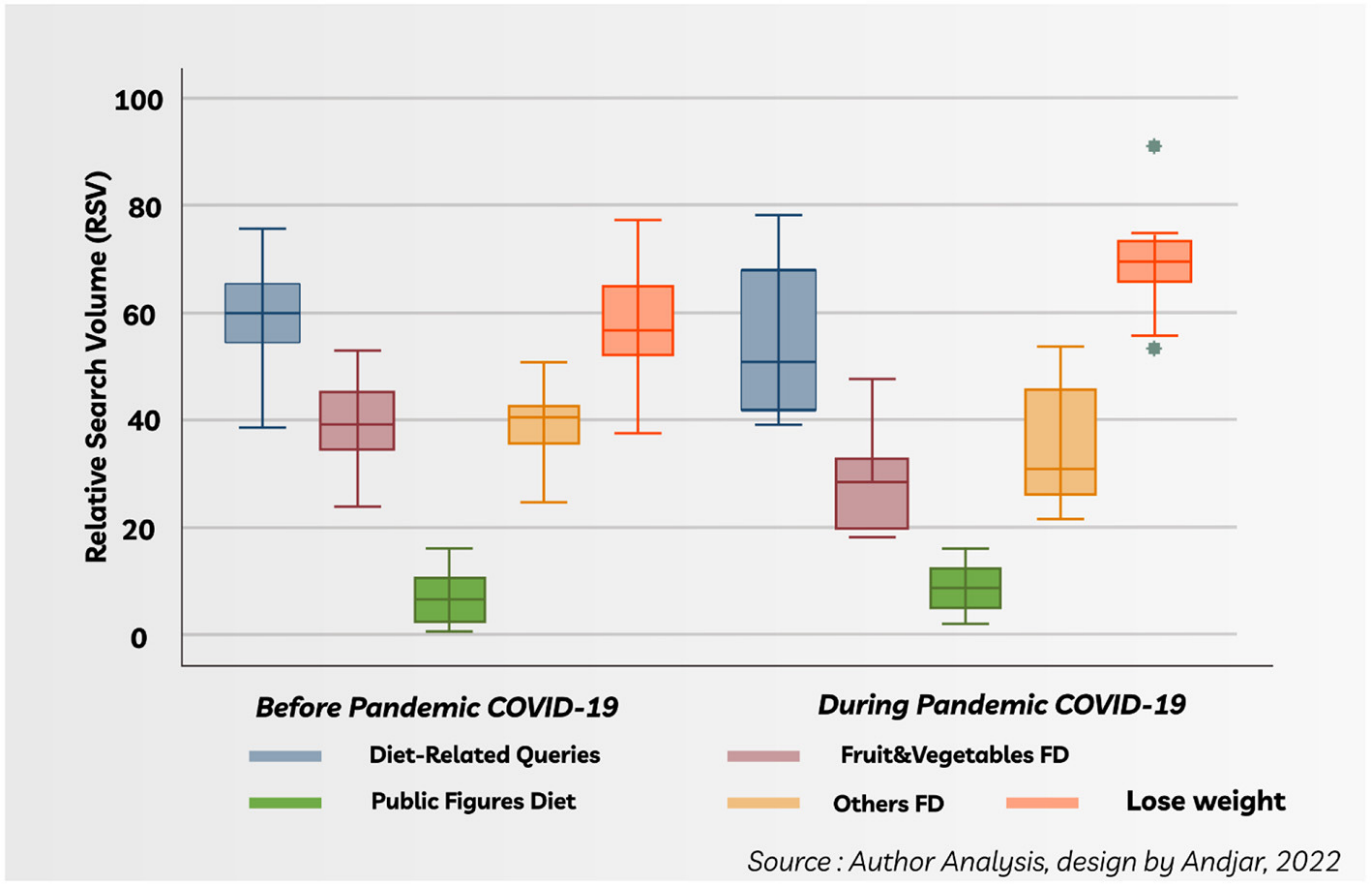
Box plots of the RSV Topics before and during the COVID-19 pandemic.

The information-seeking behavior on all topics and “lose weight” was compared before and during the pandemic. The box plots of all topics except Others FD and “lose weight” showed a slight decrease during the COVID-19 pandemic (Figure 2) but were statistically not significant (Table 3). On the other hand, the mean difference analysis showed that RSV of “lose weight” increased significantly during the COVID-19 pandemic (Table 3).

**Table 3:** The comparison of die and lose weight search behavior before and during the COVID-19 pandemic in Indonesia.

**Topic**	**Mean/Med1**	**Mean/Med2**	**Min-max1**	**Min-Max2**	**Comparison test**
Diet-related queries	60.03	50.43	38.67–75.5	38.83–78.17	1.15^a^
Fruit and vegetable FD	39.40 ± 7.87	28.22 ± 7.89	23.63–53.08	18.13–47.71	4.81^b^
Public figure FD	19.87 ± 5.56	14.01 ± 6.46	8.67–31.50	5.92–28.38	3.29^a^
Other FD	35.93 ± 6.40	38.49 ± 9.55	20.71–43.75	25.70–54.63	−1.07^2***^
“Lose weight”	58.34 ± 9.70	68.69 ± 7.72	37.25–77.25	53.20–90.75	−4.01^2**^

Source: Author Analysis, 2022.

Med, Median; Min, Minimum; Max, Maximum; 1, Before COVID-19 pandemic; 2, During COVID-19 pandemic.

Quotation marks represent queries or search terms.

a Statistical results correspond to Mann-Whitney test: ^*^p < 0.05.

b Statistical results correspond to Independent t-test: ^*^p < 0.05, ^**^p < 0.01, and ^***^p < 0.001.

Source: Author Analysis, 2022.

Further analyses were conducted on the trendline for “diet-related queries” and “lose weight” to determine the pattern of public interest in diet and losing weight. Regarding trends in diet-related queries and “lose weight” search behavior, we highlighted several interesting peaks and slopes for most topics. Marks in Figure 1 are given at the date of Eid al-Fitr (June 15, 2018; June 5, 2019; May 24, 2020, May 11, 2021), which is a Muslim holiday and the new year. Graph Figure 1 shows that the peak of diet-related keyword searches occurs consistently after Eid al-Fitr and after the new year. In contrast to the Gregorian calendar, which uses solar benchmarks, the Islamic year calendar is based on the lunar system. Although the Eid al-Fitr date changes yearly, a consistent pattern for diet-related terms spiked after Eid al-Fitr toward their peaks and then declined. The same pattern existed after the new year. The comparison of Eid al-Fitr to AM (Table 4) showed higher RSV means than the annual.

**Table 4:** The Comparison between after Eid al-Fitr and after the new year with annual diet-related queries means.

**Diet-related queries**	**2019**	**2020**	**2021**
Annual means (AM)	60.23	61.75	52.26
SD	9.54	11.50	9.43
After Eid al-Fitr means	62.46	79.96	72.57
Percentage related to AM	104%	129%	139%
January means	90.75	71.00	74.80
Percentage related to AM	151%	115%	143%

Source: Author Analysis, 2022.

The peaks after Eid al-Fitr moments showed the highest surpassed (139%) in 2021, while for new year moments, the highest surpassed (151%) was in 2019 (Table 4). This research revealed the same pattern of public interest before and during the COVID-19 pandemic.

## Discussion

Infodemiology could enhance the effectiveness of conducting disease prevention or health promotion at a more receptive time and method (16). This research revealed that public interest in the diet was not significantly different before and during a pandemic, including the pattern for heightened public interest in the diet. However, the information-seeking behavior on “lose weight” was significantly higher during the COVID-19 pandemic.

The COVID-19 pandemic dramatically impacted daily life aspects, including online interest in lifestyle behaviors (22). The higher public interest in “losing weight” could be related to a higher risk for obesity during a pandemic and the higher morbidity and mortality risk of COVID-19 infection related to obesity (21, 22).

This study identified that after Eid al-Fitr and after the new year the public interest in diet and “losing weight” were consistently in a heightened stage until the peak was reached and then declined. Eid al-Fitr is the celebration day after the fasting month of Ramadan. Community and environmental conditions could affect the increased and decreased public interest and information-seeking behavior (15, 19). This result differs from previous research conducted in four-season countries, which revealed seasonal variation. For example, research in Italy found a higher diet-related digital information seeking during the spring season (18).

Ramadan is the holy month for Muslims to fast, and they are prohibited from doing things not under Islamic teachings. Muslims should fast from dawn (fajr) to dusk in a month until the celebration day, Eid al-Fitr (31). Research revealed that fasting during Ramadan could reduce weight, but often there was an increase in body weight afterward (32). In addition, celebrations and holidays made people consume excessive food after fasting for a month (33). The surge in search terms related to weight loss at the beginning of the year is similar to a previous study in the US that showed community resolution to change lifestyle. People usually take new actions and projects to make a better life at the beginning of the year including “lose weight” and diet resolution (16).

As with previous infodemiology research on health behavior, the peak search activity could reflect the susceptible time for health promotion (30). Other than susceptible time, the implication of this study showed the potential time to prevent poor diet habits in holiday moments such as Ramadan and toward the end of the year. Dietary modification could resolve poor diet habits (34). During a celebration, people consume excessive fatty and sugary food (33). Indonesia has various traditional fruit and vegetable salads such as *pecel, gado-gado, rujak, urap, asinan*, and *lalap* which could be promoted. For alternative drinks, infused water with various healthy fruit or fruit drinks without added sugar can be an option instead of alcoholic or sweet drinks (35). Those healthy fruit and vegetable salads could be promoted to gain public interest to associate healthy food with celebration, besides more common celebration-related fatty and sugary food. Health education was important during this time. Some findings showed that nutritional education effectively increased fruit and vegetable consumption for adults and children (36, 37). The preventive action before the new year and Eid al-Fitr celebration should be practical and compelling because of the lower public interest in diet during this time. Public health policymakers must consider methods to gain public interest in a healthy diet. Some insight could be drawn from the finding of popular methods on diet.

Interest in both healthy and quick diets found in this study revealed the opportunity to offer healthier diet options. However, most diet search behavior arrived in diets promising dramatic weight loss results. It can be seen from various search terms regarding FD found since 2018. Among the popular diets in Indonesia was fruit and vegetable FD, related to limited consumption of one or several fruit and vegetables while limiting other food source consumption. This practice could lead to a heightened risk of malnutrition.

On the contrary, it indicated public interest in losing weight using natural sources with healthier expectations. The public interest encourages compelling, scientifically sound information about healthy fruit and vegetable diet, especially weight loss. Previous research found that balanced fruit and vegetable intake could support weight loss and reduce the risk of non-communicable diseases for overweight and obesity (37–39).

This research also identified several FDs related to public figures. The high public interest in FDs aligned with previous research that identified social media as powerful tools for affecting people. Public figures are influential messengers in the social media era (8). Public figures could influence people's lifestyle decisions, including their testimonials about diet (40, 41). Besides FD-related public figures, almost all FDs became well known through public figures' endorsement in various media. Publication of success story testimonials are frequently found in the place of scientific and evidence-based data (41).

Presenting a scientific body of work, including scientific and expert advice on a healthy diet, on the public stage is challenging (42). The struggle was also reflected in current research, which revealed that Indonesian official dietary guidance was not identified in the top and rising GT search terms categories. “*Isi Piringku Sekali Makan”* or “My Plating for One Mealtime” (MPFOM) is official healthy diet guidance from the Indonesian government introduced in 2017 (43). However, this health promotion was not recognized and adopted widely, unlike the previous *4 Sehat 5 Sempurna* or Four Healthy Five Perfect (FHFP) diet recommendations introduced in 1955, and now invalid because it is no longer appropriate and the information is incomplete. The recommendation was revised in 1992 with “*Pedoman Gizi Seimbang*” or nutrition balance guidance (NBG). This program which adopted the Nutrition Guide for Balanced Diet contains complete information about eating patterns. However, due to its more complex contents, ordinary people find NBG more challenging to attain, unlike its predecessor FHFP.

Previous research showed a major opportunity exists for the industry, which offers various methods to lose weight quickly and sometimes easily (44). Communication channels and others distracted information dissemination growth excessively in the last decades (40). The failure to deliver scientific healthy diet guidance to society could, in turn, heighten the risk and the prevalence of undernutrition and overnutrition in Indonesia (45). Alternative solutions for reaching wider public interest are needed. This research revealed several popular diet-related queries (“healthy diet,” “quick diet,” “diet method,” “diet menu,” and “food for diet”) and 16 popular FDs, which all use the word “diet.” The use of specific keywords in consumer campaigns had been mentioned in social engine marketing in profit and business sectors, such as in the hospitality industry (46). The use of proper keywords could gain optimum visibility in search engines (47, 48), therefore adding one of the top search terms in MPFOM could be considered because of the consistent emergence of the queries at the top and raising search terms to produce a more searchable campaign for internet users. The addition could be “diet of MPFOM,” “healthy diet of MPFOM,” or “MPFOM diet menu.” Facing distracted information growth, a healthy nutrition campaign should use the approach of utilizing search engines to achieve marketing goals. GT-based research could help policymakers understand the information-seeking behavior of the health promotion consumer (49). Regarding public figures' role in promoting a healthy diet, previous research identified three roles in various celebrity health narratives on public stages. The roles include education, inspiration, and activism (50). The public health policy maker could empower those roles in delivering public health education messages, including a scientifically based healthy and balanced diet related to weight control.

Some limitations in this research found related to the research methodology. GT did not provide information about the characteristics of Google users. Another weakness is related to the unknown actual search volume and the unknown algorithm of RSV not published by Google. Due to the segmentation internet users, it is essential to determine a study theme suitable for an infodemiological study, mainly using GT. The themes should be widely known to internet users. For example, a study about the behavior of Alzheimer's disease in older adults was improper because younger people more usually use the internet (14). This research revealed GT as a potential tool to elicit information about popular interests in diet trends. Finally, the connection between search query behavior and the actual successful public health campaign is still in the hypothetic areas. Research with actual experimentation or operational research is still needed to establish evidence-based outcomes.

## Conclusion

Diet and weight loss were popular themes among Indonesians. After Eid al-Fitr and the new year were susceptible times for promoting a healthy diet in Indonesia. On the other hand, there was a potential need before those two-point times for education in inserting traditional healthy menus among fatty and sugary menus related to holidays and celebrations. People still search for information about diet, including FD, in the COVID-19 pandemic, significantly higher for losing weight. The desire for rapid weight loss through FD needs to be resolved. We should promote healthy diets with a more compelling message and messenger. This study has validated that GT could inform the ideal timing and strategy to deliver the healthy weight loss message. In the future, it is recommended to analyze the diet and weight loss issues concerning other related health behavior, like physical activity. It is also interesting to explore diet and weight loss concerning health outcomes, such as mental health problems or non-communicable diseases like diabetes or heart disease.

## Data availability statement

The raw data supporting the conclusions of this article is available at https://drive.google.com/drive/folders/1YVgiJsTytvQfO8IRg0Mx5LPt3K1rR8Sd?usp=sharing.

## Ethics statement

Ethical review and approval was not required for the study on human participants in accordance with the local legislation and institutional requirements. Written informed consent from the participants was not required to participate in this study in accordance with the national legislation and the institutional requirements.

## Author contributions

Conceptualization, formal analysis, methodology, and writing—original draft: IR and LL. Data curation: IR. Project administration and writing—review and editing: IR, LL, AP, MK, YW, DY, and MF. Visualization: IR and AP.

## Conflict of interest

The authors declare that the research was conducted in the absence of any commercial or financial relationships that could be construed as a potential conflict of interest.

## Publisher's note

All claims expressed in this article are solely those of the authors and do not necessarily represent those of their affiliated organizations, or those of the publisher, the editors and the reviewers. Any product that may be evaluated in this article, or claim that may be made by its manufacturer, is not guaranteed or endorsed by the publisher.
